# Immune gene therapy of cancer

**DOI:** 10.3906/sag-2005-327

**Published:** 2020-11-03

**Authors:** Hakan AKBULUT

**Affiliations:** 1 Department of Basic Oncology, Ankara University Cancer Research Institute, Ankara Turkey; 2 Department of Medical Oncology, Ankara University School of Medicine, Ankara Turkey

**Keywords:** Immunotherapy, gene therapy, cancer, cytosine deaminase, granulocyte-macrophage colony-stimulating factor

## Abstract

Cancer gene therapy emerged as a promising treatment modality 3 decades ago. However, the failure of the first gene therapy trials in cancer treatment has decreased its popularity. Likewise, immunotherapy has followed a similar course. While it was a popular and promising treatment with IL-2 and interferon and cancer vaccines in the 1980s, it later lost its popularity. Immunotherapy became one of the main options for cancer treatment with the successful use of immune checkpoint inhibitors in clinics approximately 10 years ago. The success of immunotherapy has increased even more with the introduction of cancer gene therapy methods in this area. With the identification of the oncolytic herpes simplex virus and Chimeric antigen receptor (CAR) T-cells, immune gene therapy has become an essential modality in cancer treatments such as surgery, radiotherapy, chemotherapy, and targeted therapies.

## 1. Introduction

Recently, the use of new immunotherapeutic agents such as immune checkpoint inhibitors (ICIs), Chimeric antigen receptor (CAR) T-cells, and oncolytic viruses have increased median survival times in cancer. Unexpected long-term remissions with the use of several monoclonal antibodies targeting immune checkpoints such as cytotoxic T-lymphocyte-associated antigen 4 (CTLA-4), or programed death 1 (PD1)/programed death-ligand 1 (PD-L1) have raised the hope for a cure in advanced solid tumors [1]. Combining immunotherapy agents with or without cytotoxic treatments has resulted in further synergistic activity [2].

Cancer gene therapy has often been studied since the late 1990s as a promising agent in cancer treatment; however, only limited success has been achieved in humans. Talimogene laherparepvec (T-VEC), the first approved gene therapy product in cancer, has fueled gene therapy studies aiming to induce tumor-specific immunity. T-VEC is an oncolytic herpes simplex virus modified to proliferate only in tumor cells and carry the granulocyte-macrophage colony-stimulating factor (GM-CSF) gene [3].The GM-CSF produced in the tumor microenvironment induces tumor-specific immunity through the presentation of released tumor antigens by dendritic cells attracted via GM-CSF.

With the emerging role of immunotherapy in cancer, the conventional gene therapy methods that have been studied for about 30 years have started to be used to target the immune system. Like ICIs, immune targeted gene therapy approaches may yield long-term remissions in advanced cancer patients. Additionally, the combination of cytotoxic gene therapy treatments, such as suicide gene therapy and oncolytic vectors, aiming at tumor cell killing and immune-stimulation, might further increase therapeutic efficacy. In this paper, we will mainly focus on immune system targeted gene therapy.

## 2. Gene delivery systems 

Cancer gene therapy can be defined as the introduction of a therapeutic gene (transgene) into a tumor cell utilizing a delivery vehicle, called a vector. There are 2 major categories of vehicles for transporting the transgenes: viral and nonviral vectors. Nonviral vectors include the physical and chemical transfer methods of genes and bacterial and cellular vehicles. Nonviral transfer methods are usually safe and easy to use, but the transfection efficiency is usually lower than the viral vectors [4]. 

Electroporation, aiming at disrupting cell membranes using high voltage electrical pulses to facilitate the entry of DNA molecules into the cell, is a popular physical method of nonviral transport of transgenes being tested in clinical trials [5]. Likewise, nanoparticles carrying genetic material are also widely studied to deliver genes into the cells. Some bacteria, like E. *coli* and S. *typhimurium* , are used to transfer suicide genes to tumor tissues and induce host immune responses against tumors [6].Genetically engineered bacteria are usually safe and cheaper compared to viral vectors [6].However, the use of bacteria as gene therapy vehicles is limited in immune-targeted gene therapies. 

Viral vectors are widely used gene delivery vehicles in cancer treatment. The clinical trials that have been conducted so far have mainly utilized adenoviral vectors, adeno-associated viral vectors, herpes simplex viruses (HSVs), alfa viruses, retroviral vectors, and lentiviral vectors. Widely used viral vectors and their features are outlined in Table 1. Because of genomic integration, retroviral vectors and lentiviral vectors are the least preferable vectors in cancer gene therapy trials. However, lentiviral vectors are widely used for ex vivo modification of immune cells, such as DCs and T-lymphocytes [7]. Adenoviral vectors and adeno-associated vectors are commonly used to introduce therapeutic genes to tumor cells. 

**Table 1:** Viral vectors commonly used in gene therapy studies.

Viral vector	Packaging capacity (kb)	Features
Adenovirus	≤ 7.5	Transient expression in most of the cells, immunogenic.
Adeno-associated virus (AAV)	≤ 4	Long-term expression in dividing and non-dividing cells.
Herpes Simplex virus	≥ 30	Long-term expression in most of the cells; low toxicity.
Alphaviruses	≤ 7.5	Transient gene expression in most of the cells including neurons and glial cells; low immunogenicity.
Retrovirus	8	Long-term expression in dividing cells; genome integration.
Lentivirus	8	Long-term expression in both dividing and non-dividing cells; genome integration.

Adenoviruses are the most preferred viral vectors because they can express therapeutic genes episomally and have no risk of integration into the genome. First-generation adenoviral vectors have been used as carriers for the treatment of monogenic diseases by removing the E1 gene region of the vector [8]. However, first-generation adenoviral vectors are highly immunogenic, and a high prevalence of neutralizing antibodies in humans limits their clinical use. Besides, first-generation adenoviral vectors may also produce replication-competent forms during and after the production process [9]. To relieve the disadvantages mentioned above, second-generation adenoviral vectors were obtained by removing the E2 and E4 gene regions of the virus [10]. Adenoviral vectors can transduce almost all cells and are safe because they do not integrate into the genome. Likewise, transient gene expression in cells seen in adenoviral vector transductions is not an issue for cancer treatment. First and second-generation adenoviral vectors have a cargo capacity of fewer than 8 kb [10]. In order to overcome this limited cargo capacity, third-generation vectors have been obtained by further modifying the adenovirus. In this generation, all adenovirus genes have been removed except the package signals, and the cargo capacity was increased to 30 kb and called gutless vectors [11]. The vast cargo capacity of third-generation adenoviral vectors makes them attractive vehicles for cancer gene therapy. The gutless vectors have been tested in various in vitro cancer models [12]. Nevertheless, they need further improvements to increase their therapeutic potential.

Adeno-associated viruses are small nonenveloped DNA viruses from the Parvovirus group that cause latent infection in cells. They can infect both dividing and nondividing cells and integrate the genes they carry into the host genome. Because genome integration is site-specific in chromosomes, the risk of insertional mutagenesis is not as high as in retroviruses [13]. Since the transient gene expression is usually sufficient for cancer treatment, adeno-associated vectors have not been studied much in cancer treatment. In addition, their limited capacity of 4 kb cargo or less is another obstacle for the transfer of big gene constructs [14].

Alphaviruses from the Togaviridae family are used in cancer gene therapy to stimulate cytotoxic T-cell response [15]. The Semliki forest virus and Sindbis virus in this group are essential vectors that have the potential for cancer gene therapy [16].

HSVs have a high cargo capacity because of their complex genomes. As the genome size is as large as the app. 150kb, up 30 kb, genetic material can easily be loaded [17]. Not being integrated into the host genome is another advantage in terms of cancer gene therapy. Removing the immediate early genes of the virus reduces the replication capabilities to prevent the possible toxicities of the virus [17]. Modifications such as the deletion of the immediate early gene ICP47, which will enable it to reproduce only in cancer cells selectively make this vector a preferable oncolytic viral agent [18]. T-VEC, as mentioned before, acts as both an oncolytic vector and an immune-stimulating agent with its GM-CSF cargo [19].

Retroviral vectors are the second most studied vector group in cancer treatment. They are small RNA viruses and integrate into the host genome following cell entry. It is possible to load genetic material up to 10 kb by removing the capsid, reverse transcriptase, and sheath genes required for the replication of the virus [20]. As they are stably integrated into the host genome, they provide very long-term gene expression and have the potential for insertional mutagenesis. Despite their handicaps, such as low transduction efficiency and the inability to transduce nondividing cells, they are frequently used in cancer gene therapy [21].

Lentiviruses are a select group of retroviruses and are attractive because of their ability to transduce nondividing cells. They also provide long-term gene expression and low potential for inflammation. However, they can integrate into the host genome and carry the potential for insertional mutagenesis. The lentiviral vectors are mainly used to modify the T-cells [22].

## 3. Immunological targets in cancer gene therapy 

Cancer gene therapy mainly aims to transfer therapeutic genes, gene segments, or oligonucleotides either with in vivo or ex vivo approaches to the target cells. The immune system is the most crucial target for the treatment of cancer. The main immunological targets for cancer gene therapy outlined in Table 2 are cytokine/chemokine genes, tumor-associated antigens, fusion proteins, including tumor antigens, genetically modified tumor cells, or immune cells.

**Table 2:** The main immunological targets in the treatment of cancer gene therapy.

Tumor cells
Immunuostimulatory cytokines (GM-CSF, IL-12, CD40L, IL-12)
T-cells
NK cells
Suicide genes (Cytosine deaminase, Thymidine kinase)
Oncolytic vectors

The target cells are sometimes the tumor cells themselves in the immune gene therapy of cancer. In this method, gene therapy vehicles are directed against tumor cells to destroy or make them sensitive to the host immune system. Gene therapy can also target the host immune cells to make them specifically active against tumor cells. Immune cells, such as cytotoxic T-cells and dendritic cells, can also be modified exvivo utilizing gene therapy methods before administering to patients. 

### 3.1. Tumor cells as targets for immune gene therapy 

Gene therapy methods aiming at direct tumor cell killing, such as oncolytic vectors and suicide genes, can also induce tumor-specific immunity. Previously, we and other researchers have shown that tumor antigens shed from dying tumor cells may induce antitumor immunity that further improves therapeutic results [23,24]. Viruses that have cytotoxic effects against human cells were suggested as a treatment modality decades ago [25]. However, natural cytotoxic viruses (oncolytic viruses) usually failed in clinical trials. HSVs, adenoviruses, parvoviruses, and retroviruses have been modified so far to increase their therapeutic capacity and have been tested in clinical trials [26]. 

In a previous experimental tumor model, we showed that replication-competent adenoviral vectors carrying L-plastin (Lp)-driven E1a adenoviral vectors yielded significant antitumor specific immune cell killing when compared to the control ones [24,27]. Likewise, oncolytic viruses may also induce an antitumor immune response via increasing the tumor antigen shedding.

Immune gene therapy methods have been tested in various cancer cells and experimental tumor models with success. We previously designed various adenoviral vectors carrying either cytosine deaminase (CD) gene or immunostimulatory genes. Recently, we tested whether the combination of CD/5-fluorocytosine (5-FC) gene therapy, with the capability to kill tumor cells by converting 5-FC into 5-fluorouracil (5-FU) in the tumor tissue, along with an immunostimulatory GM-CSF gene, would further increase therapeutic efficacy and augment the magnitude of the antitumor immune response induced by the adjuvant effect of dying tumor cells (Figure). We constructed an adenoviral vector carrying both CD and GM-CSF genes driven by the cytomegalovirus (CMV) promoter to achieve this goal. The in vivo efficacy of the new adenoviral vector design of the bicistronic transcription unit of CD and GM-CSF and exogenous 5-FC tested in a syngeneic colon cancer model was successful [28]. Suicide gene therapy and GM-CSF induced immunity have been found to be 5 times more effective than either CD or GM-CSF alone in treatments along with the prolongation of survival times in mice. The above-mentioned adenoviral vector construct will soon be tested in a first-in-human clinical trial. 

**Figure:**
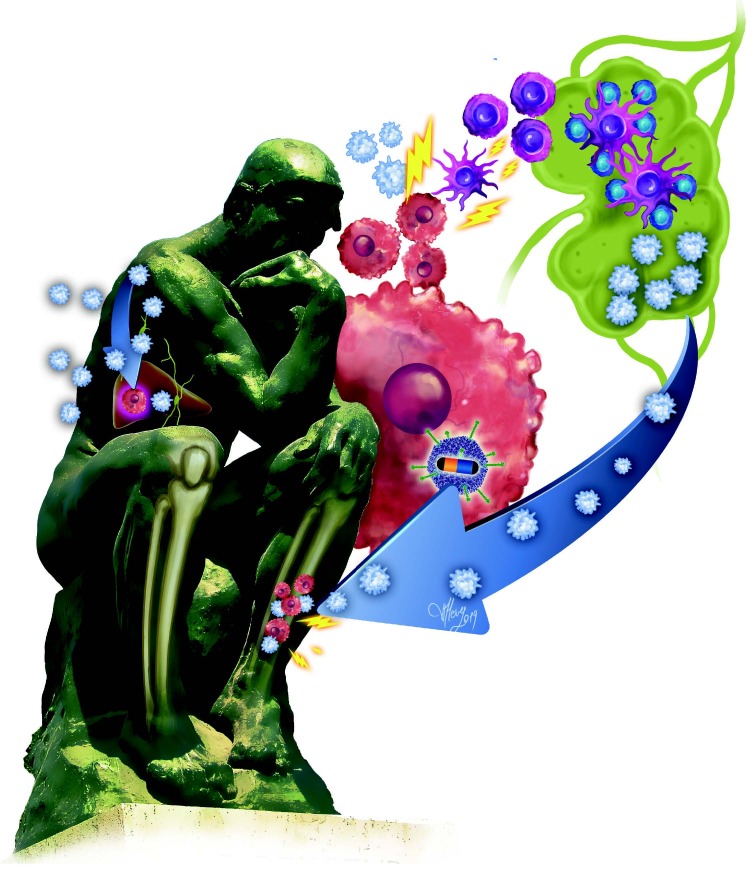
The adenoviral construct carrying cytosine deaminase and GM-CSF genes under the control of a CMV promoter produces CD and GM-CSF in tumor cells. The 5-FU produced in the tumor cell with the help of CD from 5-florocytosine, an anti-mycotic drug, kills the tumor cell and cause tumor antigen shedding. At the same time, the GM-CSF produced by the vector in the tumor cell attracts dendritic cells nearby. The immature DCs uptake tumor antigens and present to T-cells in lymph nodes. The armed T-cells then enter the systemic circulation and fight against tumor cells wherever they meet. ( : Naïve T-cell, : Armed tumor-specific T- cell, : Immature dendritic cell, : Mature dendritic cell, : Tumor cell, Adenoviral vector carrying cytosine deaminase (yellow) and GM-CSF (blue) genes.)

Combining cytotoxic treatments with immunostimulatory genes may increase therapeutic efficiency. The addition of IL-2 gene therapy to suicide gene therapies such as thymidine kinase (TK) has been shown to increase an antitumor response [29]. The immune system plays a crucial role in the development of cancer. The tumor microenvironment (TME) provides an immunosuppressive milieu [30], in which the tumor cells usually evade the immune system. Cytokines such as IL-10, VEGF, and IDO secreted by tumor cells suppress the cytotoxic T-cells [31]. Cells like MDSC, Tregs, and M2 type macrophages in the TME also suppress cytotoxic T lymphocytes. Likewise, the low pH established by the lactate from the tumor cells may further increase the immunosuppressive properties of the microenvironment [32]. Therefore, modulation of the tumor microenvironment by immunomodulatory cytokines would be beneficial.

### 3.2. Genetically modified tumor cell vaccines

Gene therapy tools have long been used to modify immune cells such as dendritic cells, cytotoxic T-cells, and autologous or allogeneic tumor cells to induce antitumor immunity. The GM-CSF gene is one of the prevalent immune cytokine genes that transduces tumor cells or dendritic cells [33]. In animal models, CT26 colon cancer cells transduced with an adenoviral vector carrying GM-CSF have induced strong antitumor immunity against tumor cells and prevented tumor regrowth [34]. This strategy has been tested in various tumor models successfully [35]. 

Clinical trials utilizing GM-CSF transduced autologous or allogeneic cancer cell vaccines have not yielded the same success rates as preclinical models. Though Tani et al. reported 2 long-term survivors out of 4 vaccinated patients with advanced renal cell carcinoma [36], no consistent results have been reported with the GM-CSF transduced autologous or allogeneic tumor cell vaccines [37]. While no objective tumor responses were seen with those vaccines, a slight increase in overall survival was noticed.

The whole of tumor cells or tumor antigens, either isolated from tumor lysates or synthetic ones, have been used in vaccination trials. Although some promising results reported in earlier trials utilized the vaccine as an adjuvant treatment, those strategies usually yielded a minimal success rate in advanced diseases [38]. Leukemia cells cannot be recognized by immune cells. Manipulation of those cells through gene therapy methods could increase their antigenicity. One such possibility is to express CD40 ligand (CD40L) on the leukemic cells to make them capable of antigen-presenting cells. The binding of CD40 expressing immune cells like T-cells and nonimmune cells induces CD95 mediated apoptosis of the leukemic cells [39]. In a phase I study of modified autologous chronic lymphocytic leukemia cells transduced with a replication-defective adenoviral vector carrying CD40L (ISF35), transduced leukemic cells made nontransduced leukemic cells present antigens and induce death-receptor induced apoptosis. They yielded clinical responses [40,41]. Later, tumor cells modified with viral vectors carrying immunostimulatory cytokine genes specifically were studied in clinical trials. In this method, the modified tumor cells behave as cellular vaccines via increasing tumor antigenicity and inducing an immune response. Comparative analysis of a modified vaccinia virus strain Ankara (MVA) encoding CD40L or TRICOM-infected chronic lymphocytic leukemia (CLL) cells showed an increased immunogenicity of those infected cells [42]. Previously, the combined expression of CD40L and IL-2, or OX40L by CLL cells transduced with adenoviral vectors, has shown antileukemic immune response [43]. Likewise, malignant B-cells from CLL patients behave as antigen-presenting cells when infected with the vectors carrying B7.1, ICAM-1, and LFA-3 costimulatory molecules [44]. A therapeutic melanoma vaccine (AGI-101H) transduced with a fusion protein consisting of soluble IL-6 receptor and IL-6 linked by a flexible peptide chain was used in the adjuvant setting in melanoma patients (44). In 2 single-arm phase II trials, AGI-101H yielded a significant prolongation in DFS and OS of stage IIB-IV resected melanoma patients compared to historical controls [45]. Accordingly, in an advanced melanoma cohort of 77 patients, the same vaccine yielded an approximate 50% disease control rate with a median OS of 17.3 months [46].

### 3.3. Immune cytokines as immune gene therapy tools

Cytokine and chemokine genes are widely studied in cancer gene therapy. GM-CSF, interferon-gamma, interferon-alpha, IL-2, IL-4, IL-24, and IL-12 are the best-known examples of cytokines used in gene therapy studies [47]. The systemic use of cytokines, such as interferon-alpha and IL-2, has caused significant toxicity in clinics, and they are no longer in use [48,49]. However, the production of those cytokines in the tumor microenvironment would decrease the toxicity. The combination of cytokine genes is also found to be effective in tumor models. Choi et al. showed that the coexpression of IL-12 and GM-CSF in the same oncolytic adenoviral vector could significantly increase antitumor immunity and could be used as a potential treatment agent in cancer [50].

Hwang et al. tested the coadministration of an adenovirus-mediated IL-12 gene transfer and a cytosine deaminase-based suicide vector followed by 5-FC treatment [51]. The coadministration of both vectors has yielded significantly higher tumor growth inhibition and prolonged median survival time in RENCA tumor-bearing mice.

4-1BB (CD137), an activation-induced costimulatory molecule expressed on activated T-cells, is an essential immune checkpoint regulator. The targeting of 4-1BB or its ligand (4-1BBL), a member of the tumor necrosis factor superfamily, may have the potential to induce antitumor immune T-cell responses. A replication-deficient adenoviral vector construct carrying 4-1BBL caused significant tumor growth inhibition in cholangiocarcinoma bearing mice [52]. Likewise, the coadministration of 2 different adenoviral vector constructs carrying either IL-12 or 4-1BBL yielded a significant antitumor T-cell response and prolonged the survival time in a mouse model bearing colon cancer (MCA26 cells) [53]. 

Chemokines recruiting the immune effector cells to the tumor microenvironment have also been used as immunostimulatory targets in gene therapy. Lapteva et al. tested the delivery of RANTES (CCL-5) via an adenoviral vector. The intratumoral injection of Ad-RANTESE1a resulted in significant tumor reduction by increasing the infiltration of macrophages, CTLs, and dendritic cells in the tumor microenvironment [54]. 

Tumor-associated antigens have long been tested as peptide vaccines for the treatment of cancer. However, the efficacy of those vaccines has been highly limited clinically. Likewise, gene therapy vectors carrying tumor-associated antigens have been tested with limited success, even in tumor models [55,56]. However, combining immune cytokine genes or checkpoint regulator genes with TAA would increase the immune response. We previously designed an adenoviral vector carrying a fusion gene encoding the CD40L and MUC1 antigens. The fusion protein yielded a significant antitumor immune response in preclinical models [57,58]. We then combined this vector vaccination with a prodrug/enzyme system. The combination therapy further increased the efficacy [57]. 

The combination of cytokine genes and TAA has also been tested in clinical trials [59]. An attenuated vaccinia vector carrying IL2 and MUC1 was reported to be effective in patients with advanced prostatic cancer [60]. Von Mehren et al. tested a vector vaccine of canarypox virus encoding B7.1 and carcinoembryonic antigen (CEA) in patients with epithelial tumors expressing CEA in a phase I trial [61]. Thirty-nine patients with CEA-expressing tumors were immunized with the vector intradermally every other week for 8 weeks. Eight out of 30 patients completing 8 vaccination cycles had a stable disease status. Although hundreds of different DNA vaccines have been tested so far, no DNA vaccine is available on the market yet. 

Although oncolytic viruses have long been studied as a cytotoxic treatment modality for cancer gene therapy, they have resulted in only limited success in clinical trials. Attempts to engineer those viruses to modulate the immune system have produced better response rates than in previous trials. HSV has been modified to selectively proliferate in tumor cells only by deleting TK, ribonucleotide reductase, or ICP34.5 genes alone or in combination [62]. However, the addition of a copy of the GM-CSF gene to the HSV vector further significantly increased therapeutic efficacy [63,64]. Also, the addition of the IL-12 gene to an oncolytic HER2-targeted HSV showed improved efficacy for metastatic tumors [65]. 

CTLA4 and PD1 are the best-known inhibitor molecules that appear on activated T-cells. Upon binding of theB7.1 or PD-L1 molecules expressed on either tumor cells or macrophages in the TME to the CTLA-4 or PD1 receptors on activated T-cells, the T-cell responses are inhibited and regressed [66]. The anti-CTL4, anti-PD1, or anti-PD-L1 antibodies, called immune checkpoint inhibitors (ICI), bind either receptor or ligands to augment the previously acquired T-cell responses against tumor cells. More than 10 monoclonal antibodies have already been approved for the treatment of various solid tumors like melanoma, lung cancer, and kidney cancer, and they are already on the market [67]. 

The manipulation of immune checkpoint ligands or receptors with gene therapy methods is also being developed. One strategy is to introduce immune checkpoint inhibitor genes to the viral vectors. Reul et al. constructed an AAV vector carrying the antihuman PD1 gene [68]. The AAV-anti-PD1 vector has successfully produced monoclonal antibodies in tumor cells, both in vitro and in vivo. Likewise, Wu et al. placed the scFv of anti-PDL1 gene into a vesicular stomatitis virus (VSV), which preferentially replicates in tumor cells [69]. The VSV carrying scFv-PDL1 has shown potentially therapeutic effects in a lung cancer mouse model with PD-L1/LLC cells. This strategy can be easily used with other checkpoint inhibitor molecules. Furthermore, the combination of ICIs with immune gene therapy tools might further increase therapeutic efficacy.

### 3.4. Genetically modified T-lymphocytes

T-cells are the primary effector cells fighting against tumor cells. Tumor-infiltrating lymphocytes (TILs) have long been suggested as a major effector population against tumor cells. However, some reports regarding the unfavorable prognostic role of the T-cell infiltrated tumor tissues have raised doubts about the use of those cells in patients [70]. Further characterization of the TILs revealed that the Treg subpopulation of T-cells in those patients resulted in an unfavorable prognosis [71]. Patients with CD8 infiltrated cells usually had a favorable prognosis [72]. The isolation of CD8+TILs from fresh tumor tissues and infusion to the patient following the expansion of the cells, so-called the adoptive transfer of T-cells, has emerged as a promising immunotherapy modality. Rosenberg et al. showed that the administration of TILs prepared from fresh surgical specimens from melanoma patients in conjunction with IL-2 and lymphodepletion yielded a 29% 5-year remission rate [73]. Adoptive transfer of TILs is found to be effective in heavily-treated patients, even with prior immunotherapies [74,75]. The number of T-cells, the proportion of CD8+ cells, and the more differentiated form of those CD8+ cells might affect therapeutic yields [76]. However, in a small, randomized study with 36 patients, the enrichment of CD8+ TILs did not increase the response rates compared to the unselected ones [77]. 

Although the adoptive transfer of TILs has resulted in promising results in melanoma, the low yield of cells isolated from fresh tumor samples and their exhausted nature make using those cells in other solid tumors a challenge. To augment the amplitude of T-cells’ activity, they are engineered to express tumor-specific T-cell receptors [78]. Clay et al. showed that the efficient transfer of the tumor-associated antigen-MART-1 reactive T-cell receptor to human lymphocytes exerted significant antitumor activity in vitro on MART-1 expressing melanoma cells [79]. Later, this strategy was translated into a clinical trial in melanoma patients by Morgan et al. [80]. Although a modest clinical activity has been achieved in that first-in-human trial, the durable objective responses in 2 patients were to herald the success of current immunotherapies. In another first-in-human trial of the T-cell receptor-targeted against E6 antigen of human papillomavirus in patients with advanced cervical cancer, sustainable objective responses were reported [81].

Due to the limited success of TILs in clinical trials, new strategies are being developed that can ensure that T-cells bind more tightly to tumor antigens. The most popular of these are CAR T-cells that are already approved for some indications in a clinical setting. The T-cell receptor (TCR) loosely binds to the target antigen, and the tumor specifically needs to be recognized by the antigen-presenting cells before they can react. Since antibody-antigen binding is more specific than TCR-antigen binding, and there is no need for prior presentation: the TCR has been replaced by the antigen-binding site of an antibody to develop potent T-cells. A chimeric antigen receptor (CAR) was obtained by using the antigen-binding region of a tumor-specific antibody fused with costimulatory molecules involved in signal transduction[82].The resultant chimeric antigen receptor gene is introduced into the T-cell through a viral vector to obtain more potent cytotoxic T-cells expressing a large number of TAA specific receptors[82]. These cells are then amplified in the laboratory and administered to patients. In the first-generation CAR T-cells, the CD3ζ chain, which plays a role in signal transduction and T-cell activation, was added next to the scFv molecule that binds to the antigen [83]. The antitumor effect of first-generation CAR T-cells was limited, and the cells underwent apoptosis after a certain period [84]. Costimulatory genes such as CD28, CD134, and 4-1BB have been added to the receptor in second and third-generation CAR T-cells [82]. Thus, the antitumoral activities of the cells increased through their ability to proliferate and secrete cytokines. Currently, CAR T-cells targeting the CD19 antigens of malignant lymphocytes have been approved for cancer treatment and have started to be used successfully in hematological malignancies, especially lymphoma and leukemia [85,86]. Although CAR T-cell therapy has limited use in solid tumors due to the shortage of unique tumor specific antigens, promising results have been reported in several recent in vivo studies [87,88]. Xia et al. have successfully used EGFR CAR T with potent and specific antitumor activity against a triple-negative breast cancer model [89]. Likewise, CEA targeted CAR T-cells are also being tested for tumors expressing CEA [90].

### 3.5. Genetically modified dendritic cells

Dendritic cells have long been used as central effector cells for cancer vaccines. The most critical antigen-presenting cells of the body are DCs. The antigen-presenting DCs could be produced through the stimulation of peripheral blood monocytes or CD34+ cells by GM-CSF and IL-4 within 3–6 days [91]. In order to further activate and to increase the maturation of the DCs against specific antigens, DCs are exposed to specific tumor antigens with either synthetic antigenic peptides or irradiated tumor cells for a few days. The tumor antigens-exposed DCs become fully activated and ready to present the tumor antigens to the immune cells. DCs have been safely tested in numerous clinical trials with some limited local inflammatory reactions and flu-like symptoms [92­–94]. However, the efficacy of those trials was modest. Specifically, the administration of DCs following surgery or cytotoxic treatments such as chemotherapy and radiotherapy is the most widely implemented strategy used to augment an immune response while the tumor burden is at the lowest level. Accordingly, cytotoxic therapy and DCs vaccines have yielded synergistic activities [94].

Dendritic cells, exvivo transduced with either immunostimulatory genes or tumor antigens and synthetic peptides, have been administered to induce an antitumor immune response. The dendritic cells activated exvivo migrate to the lymph nodes when injected subcutaneously and present tumor antigens to CD8+ cytotoxic T-cells and induce an immune response. Viral vectors carrying tumor-associated antigens have been used so far to activate DCs. We designed an adenoviral vector carrying a fusion protein of CD40L and MUC1 tumor antigen [95]. We transduced the dendritic cells with the vector carrying the CD40L-MUC1 fusion gene and tested this in a syngeneic mouse model of breast cancer intratumorally. The intratumoral injection of the dendritic cells loaded with the vaccine vector induced a potent anti-tumor CD84+ T-cell response and yielded a significant objective response. Furthermore, we also even achieved an increased immune response and tumor response, combined with the DC vaccination with suicide gene therapy of a CD/5-FC system compared to vaccination alone [95]. Adenoviral vectors, retroviral vectors, lentiviral vectors, and adenoassociated viral vectors have also been used to transduce DCs invitro [96]. A DC-based vaccine, based on the exvivo activation of blood mononuclear cells by a fusion protein consisting of GM-CSF and prostatic acid phosphatase (Provenge, Dendreon, USA), has been approved by the FDA for metastatic prostatic carcinoma [97]. Provenge has significantly extended the overall survival time for castration-resistant metastatic prostate carcinoma patients by 4 months [98]. In the case of Sipuleucel-T, as well as in most of the clinical trials with other DC-based vaccines, autologous monocyte-derived DCs (moDCs) are used. However, moDCs are not efficient enough for the recapitulation of the natural diversity of DCs. They usually mimic inflammatory DCs. Therefore, moDCs do not seem to be ideal candidates for cancer vaccination. 

The main problem with the exvivo activation of DCs is the selection of the useful cell subset of DCs. Therefore, strategies aiming at the invivo induction of DCs via powerful antigenic constructs seem much better than the ex vivo loading of DCs. The type and delivery methods of antigens used and the protocols might affect the activity of DC-based vaccines [99].

## 4. Conclusion

Immunotherapy, which started with IL-2 and interferon-alpha in the late 1980s, later increased with the use of ICIs and has become one of the main elements of cancer treatment today. Immunotherapies have provided more extended survival periods for up to more than 5 years in many metastatic tumors such as melanoma and lung cancers. A combination of immunotherapies with conventional therapies such as cytotoxic chemotherapy and radiotherapy further improved treatment outcomes. Serious side effects seen in current immunotherapeutic drugs have fueled further research efforts. The application of gene therapy methods to this field has improved the side effect profile of immunotherapy to more acceptable levels and increased treatment efficiency. Suicide gene therapies, which have cytotoxic effects on tumor cells, along with oncolytic treatments achieved with immune gene treatments, are therefore important in further increasing the success rate of cancer treatments already attained so far with ICIs and targeted therapies. 

## References

[ref1] (2019). Immune checkpoint inhibitors: The linchpins of modern immunotherapy. Immunological Reviews.

[ref2] (2018). The Antitumor Activity of Combinations of Cytotoxic Chemotherapy and Immune Checkpoint Inhibitors Is Model-Dependent. Frontiers in Immunology.

[ref3] (2016). Talimogene laherparepvec (T-Vec) for the treatment of melanoma and other cancers. Seminars in Oncology.

[ref4] (2018). Nonviral Delivery Systems for Cancer Gene Therapy: Strategies and Challenges. Current Gene Therapy.

[ref5] (2016). Clinical potential of electroporation for gene therapy and DNA vaccine delivery. Expert Opinion on Drug Delivery.

[ref6] (2014). Genetically engineered Salmonella typhimurium for targeted cancer therapy. Gene Therapy of Cancer.

[ref7] (1884). Generation of CAR-T-cells for cancer immunotherapy. Methods in Molecular Biology.

[ref8] (2017). Titration of First-Generation Adenovirus Vectors. Methods in Molecular Biology.

[ref9] (2004). Adenoviral vectors for gene replacement therapy. Viral Immunology.

[ref10] (2013). Development of an AdEasy-based system to produce first- and second-generation adenoviral vectors with tropism for CAR- or CD46-positive cells. Journal of Gene Medicine.

[ref11] (2003). Optimization of the generation and propagation of gutless adenoviral vectors. Human Gene Therapy.

[ref12] (2006). Effective high-capacity gutless adenoviral vectors mediate transgene expression in human glioma cells. Molecular Therapy.

[ref13] (2017). Recombinant Adeno-Associated Viral Integration and Genotoxicity: Insights from Animal Models. Hum Gene Therapy.

[ref14] (2015). Adeno-associated virus-mediated cancer gene therapy: current status. Cancer Letters.

[ref15] (2004). Sindbis virus - an effective targeted cancer therapeutic. Trends in Biotechnology.

[ref16] (2012). A Semliki forest virus vector engineered to express IFNalpha induces efficient elimination of established tumors. Gene Therapy.

[ref17] (2004). Replicative oncolytic herpes simplex viruses in combination cancer therapies. Current Gene Therapy.

[ref18] (2007). Properties of a herpes simplex virus multiple immediate-early gene-deleted recombinant as a vaccine vector. Virology.

[ref19] (2016). Oncolytic virus therapy: A new era of cancer treatment at dawn. Cancer Science.

[ref20] (2014). Retroviral vectors: from cancer viruses to therapeutic tools. Hum Gene Therapy.

[ref21] (2016). Retroviral vectors and transposons for stable gene therapy: advances, current challenges and perspectives. Journal of Translational Medicine.

[ref22] (2018). Clinical use of lentiviral vectors. Leukemia.

[ref23] (2011). Molecular determinants of immunogenic cell death elicited by anticancer chemotherapy. Cancer and Metastasis Reviews.

[ref24] (2009). Vector vaccination and vector targeted chemotherapy in solid tumors. Journal of BUON.

[ref25] (2015). Oncolytic viruses: a new class of immunotherapy drugs. Nature Reviews Drug Discovery.

[ref26] (2019). SnapShot: cancer immunotherapy with oncolytic viruses. Cell.

[ref27] (2003). Cytotoxic effect of replication-competent adenoviral vectors carrying L-plastin promoter regulated E1A and cytosine deaminase genes in cancers of the breast, ovary and colon. Cancer Gene Therapy.

[ref28] (2019). Bicistronic adenoviral vector carrying cytosine deaminase and Granulocyte-macrophage colony-stimulating factor increases the therapeutic efficacy of cancer gene therapy. Human Gene Therapy.

[ref29] (2010). Effect of suicide gene therapy in combination with immunotherapy on antitumour immune response & tumour regression in a xenograft mouse model for head & neck squamous cell carcinoma. Indian J Med Res.

[ref30] (2009). Tumor immunosuppressive environment: effects on tumor-specific and nontumor antigen immune responses. Expert Reviewof Anticancer Therapy.

[ref31] (2017). Exhaustion of T lymphocytes in the tumor microenvironment: Significance and effective mechanisms. Cellular Immunology.

[ref32] (2019). Lactate in the Regulation of Tumor Microenvironment and Therapeutic Approaches. Frontiers in Oncology.

[ref33] (2017). Recent progress in GM-CSF-based cancer immunotherapy. Immunotherapy.

[ref34] (1998). Irradiated tumor cells adenovirally engineered to secrete granulocyte/macrophage-colony-stimulating factor establish antitumor immunity and eliminate pre-existing tumors in syngeneic mice. Cancer Immunology Immunotherapy.

[ref35] (2016). Tumor-associated GM-CSF overexpression induces immunoinhibitory molecules via STAT3 in myeloid-suppressor cells infiltrating liver metastases. Cancer Gene Therapy.

[ref36] (2004). Phase I study of autologous tumor vaccines transduced with the GM-CSF gene in four patients with stage IV renal cell cancer in Japan: clinical and immunological findings. Molecular Therapy.

[ref37] (2006). Phase 1/2 trial of autologous tumor mixed with an allogeneic GVAX (R) vaccine in advanced-stage non-small-cell lung cancer. Cancer Gene Therapy.

[ref38] (2017). Cancer vaccines in colon and rectal cancer over the last decade: lessons learned and future directions. Expert Review of Clinical Immunology.

[ref39] (2009). Molecular mechanism and function of CD40/CD40L engagement in the immune system. Immunology Reviews.

[ref40] (2010). A phase I study of immune gene therapy for patients with CLL using a membrane-stable, humanized CD154. Leukemia.

[ref41] (2008). Active Immune Gene Therapy Using ISF35: Responses Associated with Priming for Death Receptor-Induced Apoptosis and Sensitivity to Fludarabine in Patients with CLL and Del 17p. Blood.

[ref42] (2010). Comparative analysis of MVA-CD40L and MVA-TRICOM vectors for enhancing the immunogenicity of chronic lymphocytic leukemia (CLL) cells. Leukemia Research.

[ref43] (2001). Autologous antileukemic immune response induced by chronic lymphocytic leukemia B cells expressing the CD40 ligand and interleukin 2 transgenes. Hum Gene Therapy.

[ref44] (2009). Chronic lymphocytic leukemia (CLL) cells genetically modified to express B7-1, ICAM-1, and LFA-3 confer APC capacity to T-cells from CLL patients. Cancer Immunology Immunotherapy.

[ref45] (2012). Kapcinska Met al. Long-term survival of high-risk melanoma patients immunized with a Hyper-IL-6-modified allogeneic whole-cell vaccine after complete resection. Expert Opinionon Investigational Drugs.

[ref46] (2015). Whole Cell Therapeutic Vaccine Modified With Hyper-IL6 for Combinational Treatment of Nonresected Advanced Melanoma. Medicine (Baltimore).

[ref47] (2010). Advances in viral-vector systemic cytokine gene therapy against cancer. Vaccine.

[ref48] (2010). Interferon alfa-2a versus combination therapy with interferon alfa-2a, interleukin-2, and fluorouracil in patients with untreated metastatic renal cell carcinoma (MRC RE04/EORTC GU 30012): an open-label randomised trial. Lancet.

[ref49] (2008). Adjuvant therapy with pegylated interferon alfa-2b versus observation alone in resected stage III melanoma: final results of EORTC 18991, a randomised phase III trial. Lancet.

[ref50] (2012). Strengthening of antitumor immune memory and prevention of thymic atrophy mediated by adenovirus expressing IL-12 and GM-CSF. Gene Therapy.

[ref51] (2005). Adenovirus-mediated interleukin-12 gene transfer combined with cytosine deaminase followed by 5-fluorocytosine treatment exerts potent antitumor activity in Renca tumor-bearing mice. BMC Cancer.

[ref52] (2003). A novel adenovirus expressing human 4-1BB ligand enhances antitumor immunity. Cancer Immunology Immunotherapy.

[ref53] (2000). Immunomodulatory gene therapy with interleukin 12 and 4-1BB ligand: long- term remission of liver metastases in a mouse model. Journal of National Cancer Institute.

[ref54] (2009). Targeting the intratumoral dendritic cells by the oncolytic adenoviral vaccine expressing RANTES elicits potent antitumor immunity. Journal of Immunotherapy.

[ref55] (2000). Antitumor efficacy of tumor-antigen-encoding recombinant poxvirus immunization in Dunning rat prostate cancer: implications for clinical genetic vaccine development. World Journal of Urology.

[ref56] (2013). TAA/ecdCD40L adenoviral prime-protein boost vaccine for cancer and infectious diseases. Cancer Gene Therapy.

[ref57] (2006). Vector prime/protein boost vaccine that overcomes defects acquired during aging and cancer. Journal of Immunology.

[ref58] (2010). Addition of adenoviral vector targeting of chemotherapy to the MUC-1/ecdCD40L VPPP vector prime protein boost vaccine prolongs survival of mice carrying growing subcutaneous deposits of Lewis lung cancer cells. Gene Therapy.

[ref59] (2013). Gene therapy clinical trials worldwide to 2012 - an update. Journal of Gene Medicine.

[ref60] (2003). Phase I immunotherapy with a modified vaccinia virus (MVA) expressing human MUC1 as antigen-specific immunotherapy in patients with MUC1-positive advanced cancer. Journal of Gene Medicine.

[ref61] (2000). Pilot study of a dual gene recombinant avipox vaccine containing both carcinoembryonic antigen (CEA) and B7.1 transgenes in patients with recurrent CEA-expressing adenocarcinomas. Clinical Cancer Research.

[ref62] (2010). Development of a regulatable oncolytic herpes simplex virus type 1 recombinant virus for tumor therapy. Journal of Virology.

[ref63] (2010). OPTIM trial: a Phase III trial of an oncolytic herpes virus encoding GM-CSF for unresectable stage III or IV melanoma. Future Oncology.

[ref64] (2014). Oncolytic viral therapy with a combination of HF10, a herpes simplex virus type 1 variant and granulocyte-macrophage colony-stimulating factor for murine ovarian cancer. International Journal of Cancer.

[ref65] (2018). A fully-virulent retargeted oncolytic HSV armed with IL-12 elicits local immunity and vaccine therapy towards distant tumors. PLoS Pathology.

[ref66] (2018). Fundamental Mechanisms of immune checkpoint blockade therapy. Cancer Discovery.

[ref67] (2018). Immune checkpoint inhibitors: recent progress and potential biomarkers. Experimental Molecular Medicine.

[ref68] Tumor-specific delivery of immune checkpoint inhibitors by engineered AAV vectors.

[ref69] (2019). in Oncology.

[ref70] (2019). A novel oncolytic virus engineered with PD-L1 scFv effectively inhibits tumor growth in a mouse model. Cellular and Molecular Immunology.

[ref71] (1992). Tumour infiltrating lymphocytes as an independent prognostic factor in transitional cell bladder cancer. European Journal of Cancer.

[ref72] (2015). Low infiltration of peritumoral regulatory T-cells predicts worse outcome following resection of colorectal liver metastases. Annals of Surgical Oncology.

[ref73] (2020). TNFR2+ TILs are significantly associated with improved survival in triple-negative breast cancer patients. Cancer Immunology Immunotherapy.

[ref74] (2011). Durable complete responses in heavily pretreated patients with metastatic melanoma using T-cell transfer immunotherapy. Clinical Cancer Research.

[ref75] (2013). Adoptive transfer of tumor-infiltrating lymphocytes in patients with metastatic melanoma: intent-to-treat analysis and efficacy after failure to prior immunotherapies. Clinical Cancer Research.

[ref76] (2017). Treatment of metastatic uveal melanoma with adoptive transfer of tumour-infiltrating lymphocytes: a single-centre, two-stage, single-arm, phase 2 study. Oncology.

[ref77] (2012). Specific lymphocyte subsets predict response to adoptive cell therapy using expanded autologous tumor-infiltrating lymphocytes in metastatic melanoma patients. Clinical Cancer Research.

[ref78] (2013). Randomized selection design trial evaluating CD8+-enriched versus unselected tumor-infiltrating lymphocytes for adoptive cell therapy for patients with melanoma. Journal of Clinical Oncology.

[ref79] (2017). Safety and persistence of WT1-specific T-cell receptor gene-transduced lymphocytes in patients with AML and MDS. Blood.

[ref80] (1999). Efficient transfer of a tumor antigen-reactive TCR to human peripheral blood lymphocytes confers anti-tumor reactivity. Journal of Immunology.

[ref81] (2006). Cancer regression in patients after transfer of genetically engineered lymphocytes. Science.

[ref82] (2019). T-Cell receptor gene therapy for human papillomavirus-associated epithelial cancers: A First-in-human, phase I/II study. Journal of Clinical Oncology.

[ref83] (2019). Generation of CART-Cells for cancer immunotherapy. Methods in Molecular Biology.

[ref84] (2018). Programming CAR-T-cells to kill cancer. Nature Biomedical Engineering.

[ref85] (2017). The clinical efficacy of first-generation carcinoembryonic antigen (CEACAM5)-specific CAR T-cells is limited by poor persistence and transient pre-conditioning-dependent respiratory toxicity. Cancer Immunology Immunotherapy.

[ref86] (2018). A Phase I/IIa trial using CD19-targeted third-generation CAR T-cells for lymphoma and leukemia. Clinical Cancer Research.

[ref87] (2018). Determinants of response and resistance to CD19 chimeric antigen receptor (CAR) T-cell therapy of chronic lymphocytic leukemia. Nature Medicine.

[ref88] (2019). CAR T-cells for solid tumors: New strategies for finding, infiltrating, and surviving in the tumor microenvironment. Frontiersin Immunology.

[ref89] (2020). Challenges and prospects of chimeric antigen receptor T-cell therapy for metastatic prostate cancer. European Urology.

[ref90] (2020). EGFR‐targeted CAR‐T-cells are potent and specific in suppressing triple‐negative breast cancer bothin vitroandin vivo. Clinical & Translational Immunology.

[ref91] (2017). T-cells targeting solid tumors: carcinoembryonic antigen (CEA) proves to be a safe target. Cancer Immunol Immunotherapy.

[ref92] (2020). Dendritic cells in anticancer vaccination: rationale for ex vivo loading or in vivo targeting. Cancers.

[ref93] (2017). Phase I Trial of Intratumoral Injection of CCL21 Gene-modified dendritic cells in lung cancer elicits tumor-specific immune responses and CD8(+) T-cell infiltration. Clinical Cancer Research.

[ref94] (2019). Predicted markers of overall survival in pancreatic cancer patients receiving dendritic cell vaccinations targeting WT1. Oncology.

[ref95] (2018). Randomized controlled phase III trial of adjuvant chemoimmunotherapy with activated cytotoxic T-cells and dendritic cells from regional lymph nodes of patients with lung cancer. Cancer Immunology Immunotherapy.

[ref96] (2006). Antitumor immune response induced by i.t. injection of vector-activated dendritic cells and chemotherapy suppresses metastatic breast cancer. Molecular Cancer Therapy.

[ref97] (2003). Viral vectors for dendritic cell-based immunotherapy. Current Topics in Microbiological Immunology.

[ref98] (2011). Immunotherapy for the treatment of prostate cancer. Nature Reviews Clinicl Oncology.

[ref99] (2010). Sipuleucel-T immunotherapy for castration-resistant prostate cancer. New England Journal of Medicine.

[ref100] (2016). Dendritic cell-based immunotherapy: State of the art and beyond. Clinical Cancer Research.

